# Analyzing the efficiency of short-term air quality plans in European cities, using the CHIMERE air quality model

**DOI:** 10.1007/s11869-016-0427-y

**Published:** 2016-09-10

**Authors:** P. Thunis, B. Degraeuwe, E. Pisoni, F. Meleux, A. Clappier

**Affiliations:** 1 European Commission, Joint Research Centre (JRC), Directorate for Energy, Transport and Climate, Air and Climate Unit, Via E. Fermi 2749, Ispra, I-21027 VA Italy; 2 Institut National de l’Environment Industriel et des Risques, Verneuil en Halatte, France; 3 Laboratoire Image Ville Environnement, Université de Strasbourg, Strasbourg, France

**Keywords:** Forecast, Air quality modeling, Emission scenarios, Air quality planning, PM_10_, NO_2_

## Abstract

Regional and local authorities have the obligation to design air quality plans and assess their impacts when concentration levels exceed the limit values. Because these limit values cover both short- (day) and long-term (year) effects, air quality plans also follow these two formats. In this work, we propose a methodology to analyze modeled air quality forecast results, looking at emission reduction for different sectors (residential, transport, agriculture, etc.) with the aim of supporting policy makers in assessing the impact of short-term action plans. Regarding PM_10_, results highlight the diversity of responses across European cities, in terms of magnitude and type that raises the necessity of designing area-specific air quality plans. Action plans extended from 1 to 3 days (i.e., emissions reductions applied for 24 and 72 h, respectively) point to the added value of trans-city coordinated actions. The largest benefits are seen in central Europe (Vienna, Prague) while major cities (e.g., Paris) already solve a large part of the problem on their own. Eastern Europe would particularly benefit from plans based on emission reduction in the residential sectors; while in northern cities, agriculture seems to be the key sector on which to focus attention. Transport is playing a key role in most cities whereas the impact of industry is limited to a few cities in south-eastern Europe. For NO_2_, short-term action plans focusing on traffic emission reductions are efficient in all cities. This is due to the local character of this type of pollution. It is important, however, to stress that these results remain dependent on the selected months available for this study.

## Introduction

Where and whenever EU air quality limit thresholds are exceeded, authorities have a formal obligation to design air quality plans and assess their impacts on concentration levels. For PM_10_ and NO_2_, both short-term (daily for PM_10_ and hourly for NO_2_) and long-term (yearly) limits exist (EU 2008). To achieve these goals, national and regional authorities have developed and applied a variety of approaches, where models play a key role (Menut et al. 2013). Air quality models indeed represent the ideal instrument to design and assess the impacts of air quality plans (Carnevale et al. 2014) as they allow testing easily various policy options.

While many studies have been devoted to the analysis of long-term air quality plans (i.e., see Carnevale et al. 2008, Cuvelier et al. 2007, Miranda et al. 2015), less have addressed the problem of pollution episodes (Rezer and Juda-Rezler, 2016, Tsakiri and Zurbenko, 2011). These pollution episodes are, however, extremely visible and catch the attention of the media. Although authorities often act and apply a set of measures (alternate traffic, reduced speed limits, etc.) the efficiency of these measures is often questioned (Lasry et al. 2007). Not only the measures themselves but also their timing of application as well as the spatial area over which these measures should be applied remain an open question.

In the frame of MACC, MACC-II, and MACC-III EU projects, specific modeling products are being developed with the intention of supporting policy makers in the design of relevant policy responses to prevent severe air pollution episodes. One of these products is referred to as the “green scenario toolbox” (http://macc.copernicus-atmosphere.eu/services/aqac/policy_interface/green_scenarios/control_scenarios/). It provides daily regular information on the expected effect that short-term theoretical measures on various emission sources may have on the forecasted pollution episodes (control scenario).

In this work, the MACC scenarios will be analyzed with a methodology (Thunis and Clappier 2014, Thunis et al. 2015) developed in the frame of the forum for air quality modeling (FAIRMODE, http://fairmode.jrc.ec.europa.eu/). The main aim of FAIRMODE is to provide harmonized air quality modeling methodologies and ensure they are fit for purpose. Methodologies specifically designed for the dynamic evaluation of air quality models have been developed and are well fit to analyze the model responses to the MACC green scenarios.

The main aim of this work is to assess the efficiency of short-duration air quality plans in different European cities and identify the priority activity sectors to abate in each of these cities, using/testing the planning indicators developed in the frame of FAIRMODE. We look at the added value of (1) coordinating actions among cities and (2) extending the duration of the air quality plans (AQP) from 1 to 3 days (i.e., applying emission reduction measures for 1 day only or for 3 consecutive days). These aspects will be treated for both PM_10_ and NO_2_, although the scenarios were primarily designed to target particulate matter.

We will start by describing briefly the methodology and model set-up. Then, we will split the analysis in two parts: 1- and 3-day-long action plans. Finally, we address the potential sources of uncertainty and assumptions underlying this analysis.

## Methodology

The methodology used in this work follows the approach proposed by Thunis and Clappier (2014) and Thunis et al. (2015), where potency and potential indicators are proposed to analyze the model responses to changes in emission input. As we focus on the efficiency of emission reduction abatements on air quality in cities and wish to distinguish the contributions of emission sources according to the duration (number of days) of an air quality plan, we first mathematically decompose the concentration observed in a city on a given day, *i*, in its different components, as follows:

1 $$ {C}_{\mathrm{city}}^i={C}_{bg}^{nat,i}+{\displaystyle \sum_{d=-\infty}^{i-{D}_{AQP}}\varDelta {C}_{100\%}^{\mathrm{ant},d}}+{\displaystyle \sum_{d=i-{D}_{AQP}+1}^i\varDelta {C}_{100\%}^{\mathrm{ant},d}} $$

where ${C}_{bg}^{nat}$ represents the natural background (wind-blown dust, Saharan dust event, etc.) and the two other terms represent the anthropogenic contributions split into a short (*D*_AQP_ days) and a longer term (contributions before the AQP is in action) where *D*_AQP_ represents the number of days during which emission reduction are applied. In this work, *D*_AQP_ will vary in practice from 1 to 3 days. The delta contributions are intended as concentration changes, resulting from a reduction of 100 % of the anthropogenic emission amount over a given day. The longer-term contribution will be referred here as the anthropogenic background (${C}_{bg}^{\mathrm{ant},i}={\displaystyle \sum_{d=-\infty}^{i-{D}_{AQP}}\varDelta {C}_{100\%}^{\mathrm{ant},d}}$) as this represents the emission fraction that will not impact the city concentrations in the short term. This may result from either long horizontal distance covered (e.g., distant cities), long vertical dispersion delay (e.g., elevated point sources), or slow chemical processes. It is important to note that this anthropogenic background will tend to be reduced with longer air quality plan, compensated by increased local effects. On the other hand, the short-term contribution can be split in terms of its composing emission sectors as follows:

2 $$ {C}_{\mathrm{city}}^i={C}_{bg}^{nat,i}+{C}_{bg}^{\mathrm{ant},i}+{\displaystyle \sum_{d=i-{D}_{AQP}+1}^i\left[{\displaystyle \sum_{s=1}^{N_{\sec t}}\varDelta {C}_{100\%}^{s,d}}+{f}_{NL}^d\right]} $$

The term (${\displaystyle \sum_{s=1}^{N_{\sec t}}\varDelta {C}_{100\%}^{s,d}}$) sums up the city contributions in terms of their activity sectors (e.g., transport, agriculture, etc.) for a given day (*d*) while the term *f* ^*d*^_NL_ represents the possible non-linear interactions among sectors (indeed the impact of reducing separately two sectors does not necessarily equal the impact of simultaneously reducing both sectors). In the following, we will assume that these non-linear interaction terms can be neglected. The validity of this assumption is discussed in the “Uncertainties and limitations of the approach” section. If we divide Eq. (2) by the concentration, we obtain:

3 $$ 1=\frac{C_{bg}^{nat,i}}{C_{\mathrm{city}}^i}+\frac{C_{bg}^{\mathrm{ant},i}}{C_{\mathrm{city}}^i}+{\displaystyle \sum_{d=i-{D}_{AQP}-1}^i{\displaystyle \sum_{s=1}^{N_{\sec t}}\frac{\varDelta {C}_{100\%}^{s,d}}{C_{\mathrm{city}}^i}}} $$

where all contributions are now expressed in relative terms. The third term on the right side of this equation is now expressed as a sum of daily and sectorial detailed relative potentials. We refer the reader to Thunis et al. (2015) for more details.

To compute all the terms of the previous equation, the CHIMERE chemistry-transport model (Menut et al. 2013) has been used. Because the CHIMERE simulations are obtained with partial emission reductions (*α* = 30 %), we make the following linear assumption:4


In the following analysis, we will focus on term *C* and assess (1) how this term varies with the duration of the air quality plan (*D*_AQP_) and (2) how the sectoral contributions differ in terms of the AQP duration.

It is important to stress that relation (2) is day dependent; implying that the calculated impacts on two different days of an equivalent duration emission reductions will not be comparable. One of the main reasons behind this daily dependency lies obviously in the meteorology which can either exacerbate or inhibit the impact of the emission reduction on the concentrations. For the same reason, the differences in impacts between air quality plans of different durations cannot be only attributed to emissions. To prevent this problem, all results are averaged over a large number of days so that the meteorological variability is smoothed out. The reader is referred to the Annex for more details regarding this averaging step. The previous equation now becomes:


5


in which all *C* notations (i.e., without their daily indices) denote concentrations averaged over a long (several months) time period. The summation can now be expressed from *d* = 1 to *D*_AQP_ as it becomes day independent.

Before analyzing the results, in the following section, a brief description of the model set-up is provided as well as some explanations on how the potential indicators are constructed from the available scenarios.

## Model set-up and emission scenarios

The CHIMERE chemical transport model (Menut et al. 2013) has been used in this work to perform scenario simulations over the entire European domain, in the frame of MACC-III project (for more details on the model set-up, input data and scenarios, we refer to http://www.gmes-atmosphere.eu/services/aqac/policy_interface/green_scenarios/). The CHIMERE model has been extensively evaluated to show its ability to forecast O_3_, PM_10_, and NO_2_ concentrations (Rouil et al., 2009; Honoré et al. 2008; Marécal et al., 2015) and no further validation is provided in this work since the main interest is the analysis of the model responses to emission scenarios and their possible implications in terms of policy.

**Fig. 1:**
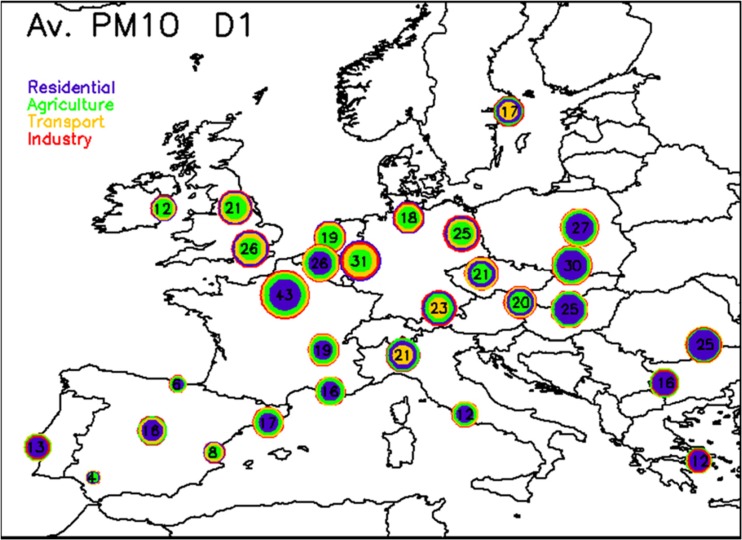
Map of relative potentials (i.e., ∆*C*/*αC*) at *D*_AQP_ = 1 for PM_10_. The *circled area* is proportional to the potential with the most important contributors placed from center to outwards. The four activity sectors are represented by *different colors*. The *number in each circle* is the overall potential (i.e., corresponding to all sectors reduced simultaneously)

All simulations have been performed (see Fig. 1 for the domain definition) with a spatial resolution of 0.5°× 0.5° (~50 km). This coarse spatial resolution only allows for a “background concentrations” analysis. Because the CHIMERE simulations used in the present analysis were performed in a pre-operational phase of the project, only 55 days within the 5-month period were available, considering 4 days in January, 8 in February, 16 in March, 17 in April, and 10 in May. Despite these limitations, the application can serve as an interesting case study, which could be repeated when higher spatial/temporal resolution data will be available.

Each day, a reference run is performed from the day before up to 3 days ahead. Then, the pollutant concentrations obtained after 1 day are used to initiate the four scenario runs, each lasting 3 days. For each day during this time period, emission reductions are applied to one of the following activity sector: agriculture (AGR – SNAP 10), transport (TRA – SNAP 7), residential heating (RES – SNAP 2), and industry (IND – SNAPs 1, 3, 4, 5, 6). For each of these sectors, emissions are reduced by an amount of 30 % over the entirety of the European modeling domain. Although this abatement level has been chosen as representative of feasible reductions, it remains theoretical and is not related to any specific measures. At the beginning of each day, these emissions are reduced over a 24 h (*D*_AQP_ = 1), 48 h (*D*_AQP_ = 2), and 72 h (*D*_AQP_ = 3) time period (see Table 1).

**Table 1:** Description of the emission scenarios. The table is valid for all four sectors reduced in this analysis

	24 h (*D*_AQP_ = 1)	48 h (*D*_AQP_ = 2)	72 h (*D*_AQP_ = 3)
Sector (AGR, TRA, DOM, IND)	−30 %		
	−30 %	−30 %	
	−30 %	−30 %	−30 %

As mentioned earlier, this work focuses on the results obtained over selected cities where exceedances of the threshold values (i.e., yearly averages above 50 and 40 μg/m^3^ for PM_10_ and NO_2_, respectively) are observed. As not all EU cities can be considered, we therefore performed the analysis for a selection of 30 major European cities distributed in the various countries. Urban grid-cells in and around the selected city centers are used to extract the time series of interest.

The results are obviously highly dependent on the quality of the emission inventory underlying the model simulations. In the current configuration, anthropogenic emissions are based on the MACC-TNO emission inventory (Kuenen et al. 2014) while the meteorology is based on operational forecasts from the IFS model (ECMWF, Dee et al. 2011). A specific processing modulates the emissions from the residential sector, to take into account more accurately the enhancement of wood consumption in case of extremely low temperature (Terrenoire et al. 2015).

It is important to note that the emission scenarios considered here do not cover the entirety of the anthropogenic emission sources. Transport other than road traffic (i.e., off-road, shipping, air transport, etc.) are, for example, not covered. To reflect the fact that some sources are explicitly considered in the emission scenario simulations and others not, we further split term C into two parts: controlled sources (term *C* Eq. 6) and uncontrolled sources (term *D* Eq. 6):


6


In the following two sections, we will investigate how this term *C* based on relative potentials reacts (a) in terms of the duration of the imposed emission reductions, (b) in terms of the pollutant, and (c) in terms of the city characteristics.

## Efficiency of “24 h” action plans

The efficiency of short-term (24 h, i.e., *D*_AQP_ = 1) air quality plans is shown in Fig. 1. It provides an overview of the magnitude of the relative potentials reached in the different EU cities. The area contained by the outer circle of each city is proportional to the overall relative potential (number written inside the circle). It gives the relative change in concentration that would be obtained when reducing fully all four main activity sectors (transport, residential, agriculture, and industry) by 100 %. The inner circles provide similar information but for specific activity sectors. The contributions of the different sectors are ranked in terms of their relative importance from center to outwards.

First, it is important to stress the strong link which exists between the temporal and the spatial dimensions of air quality plans. Indeed, emission reductions applied in areas located far away from the city will not impact the city, because of the too limited time allowed for advection and/or transformation. In other words, short-term (24 h) action plans can also be seen and understood as local action plans, with local to be understood as a daily impact radius. This impact radius will differ from city to city depending on the locally prevailing meteorological conditions (horizontal advection as well as vertical diffusion) and the speed of chemical transformations.

We first note that the relative potentials obtained after a 24-h emission reduction (Fig. 1) are well below 100 %, value representative of a full potential. This is due to various possible causes which can be identified from previous equations:

- Natural sources (term *A* in Eq. 6) contribute to the overall concentration level and will not be affected by the emission scenarios considered in our analysis.
- The background city level (term *B* in Eq. 6) includes city trans-boundary impacts. These impacts are not seen in the potential terms which only account for the local emission reductions effects because of the too limited time of the abatement measures plan (24 h) which does not allow for long distance transport.
- Not all anthropogenic sources are considered in the scenarios (term *D* in Eq. 6). This is the case of shipping or air transport for example. These sectors might be contributing significantly in and around some cities.
- Positive non-linearities (last term in Eq. 1) might occur which are not considered in this study.

From Fig. 1, we note that the maximum overall potentials occur in the Paris region (43 %), the Benelux (26 %) and the Ruhr area (31 %), as well as in some Eastern countries (30 %). These cities (and surrounding regions) are therefore the places in Europe where air quality plans are most efficient even if those plans are characterized by a limited spatial and temporal extension. It is important to keep in mind that these numbers depend on (1) meteorological conditions (e.g., the mild 2015 winter in Western Europe has some impact on the relative importance of residential heating) and (2) the repartition of available days within the 5-month analyzed period (e.g., agriculture has larger emissions during the March–April period).

The main contributor (highest relative potential) in most of the northern cities (and surrounding regions) is agriculture (e.g., London, Amsterdam, Hamburg, Vienna) while residential heating plays the key role in most of the Eastern countries but also in France and in the Iberian Peninsula. This issue is also linked to the days selected for the analysis (between January and May). Transport is the key contributor in Milan, Munich, and Stockholm.

One possible explanation for the large contribution of residential heating in some cities for short-term action plans is related to the larger fraction of primary particulate emissions for this sector (especially wood burning). This emission indeed directly contributes to the PM concentrations, in contrast to the gas emission precursors (e.g., NO_x_, VOC, or NH_3_) that require chemical transformations (and therefore more time) before generating particulate matter.

Agriculture plays a key role in most of the northern cities (even if not dominating in all of them). Since agriculture emissions are most often located outside the city center, this result implies that 24 h already represents a sufficiently long time to include impacts from the city surrounding areas in these northern locations. The role of industry is limited to a few cities in Germany. One of the reasons for the limited impact of industry is the fact that most industrial emissions are released at higher heights, away from city centers. Emissions then necessitate time to be mixed down to the city level and transported there to impact the surface concentrations.

**Fig. 2:**
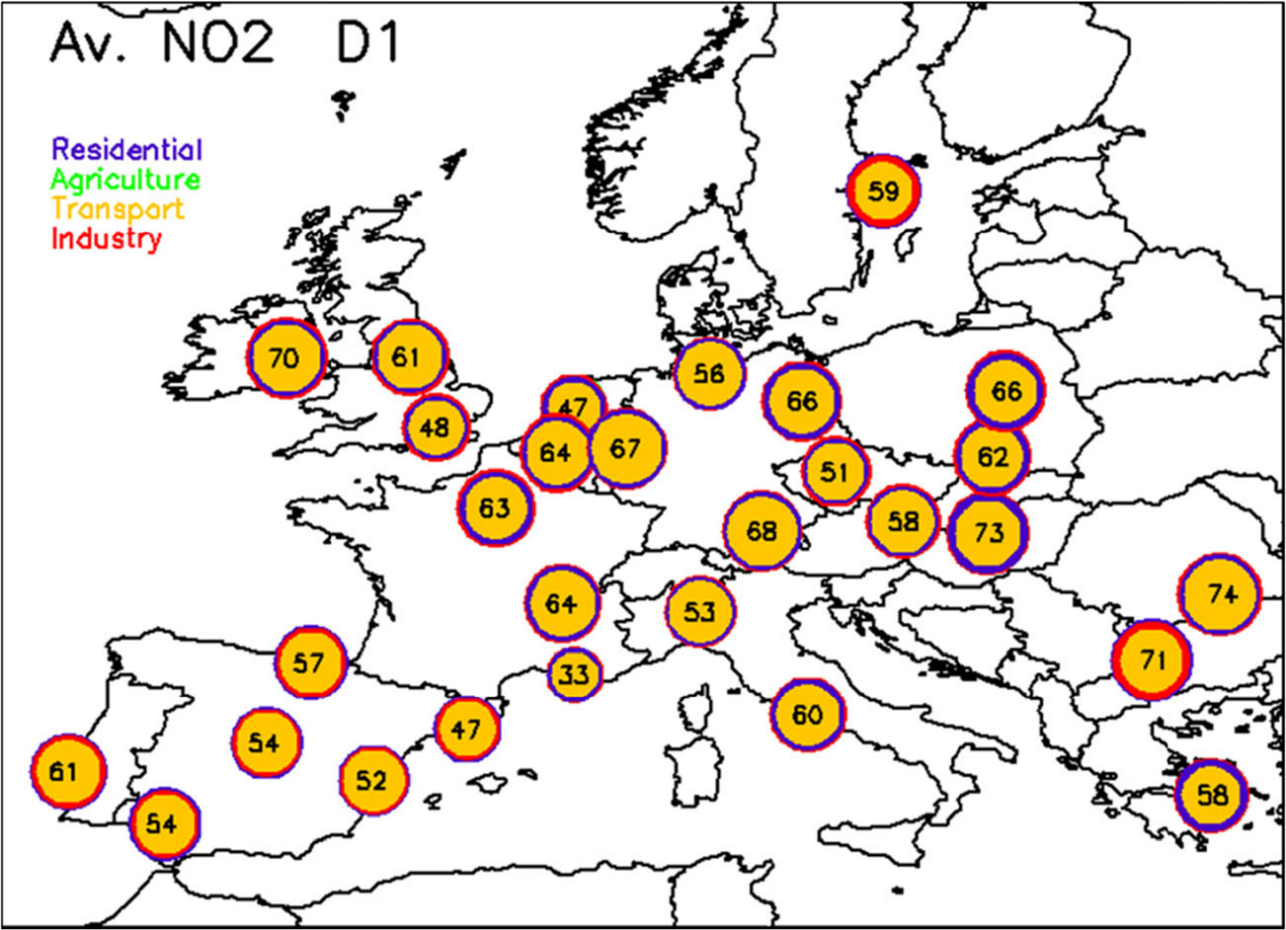
Same as Fig. 1 but for NO_2_

With the exception of a few major cities (like Paris), 24-h action plans are not very efficient for PM_10_. Similar conclusions can be drawn for PM_2.5_ (not shown). Before analyzing in the next section, the differences in terms of impact between 24 and 72-h action plans, we first repeat this analysis for NO_2_ (Fig. 2). All potentials are significantly larger and exhibit a quite homogeneous range of values across Europe (between 50 and 75 %). Transport is the major contributor in all cities while industry plays some role in Sofia and Stockholm, residential heating also plays some role in the Eastern countries but it is not dominant. Contrary to PM_10_ and PM_2.5_, local action plans (with limited time span) already provide efficient solutions with transport being the sector to target in the first place for all cities.

## Efficiency of “72 h” action plans

**Fig. 3:**
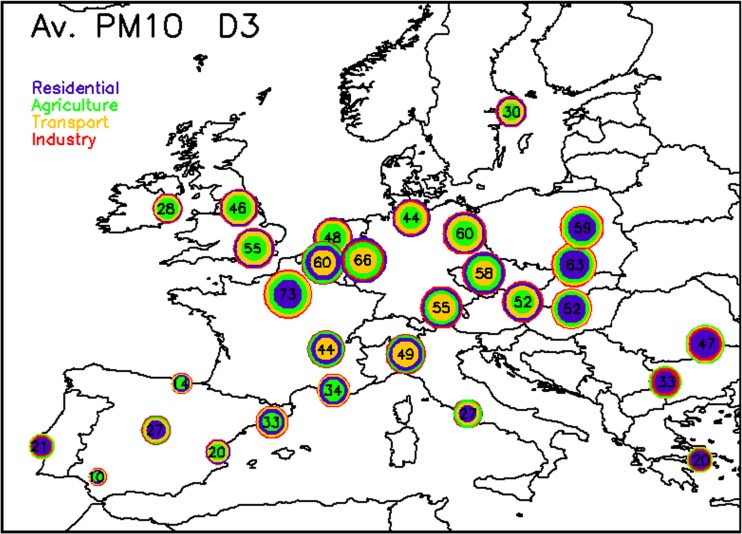
Same as Fig. 1 but for *D*_AQP_ = 3

**Fig. 4:**
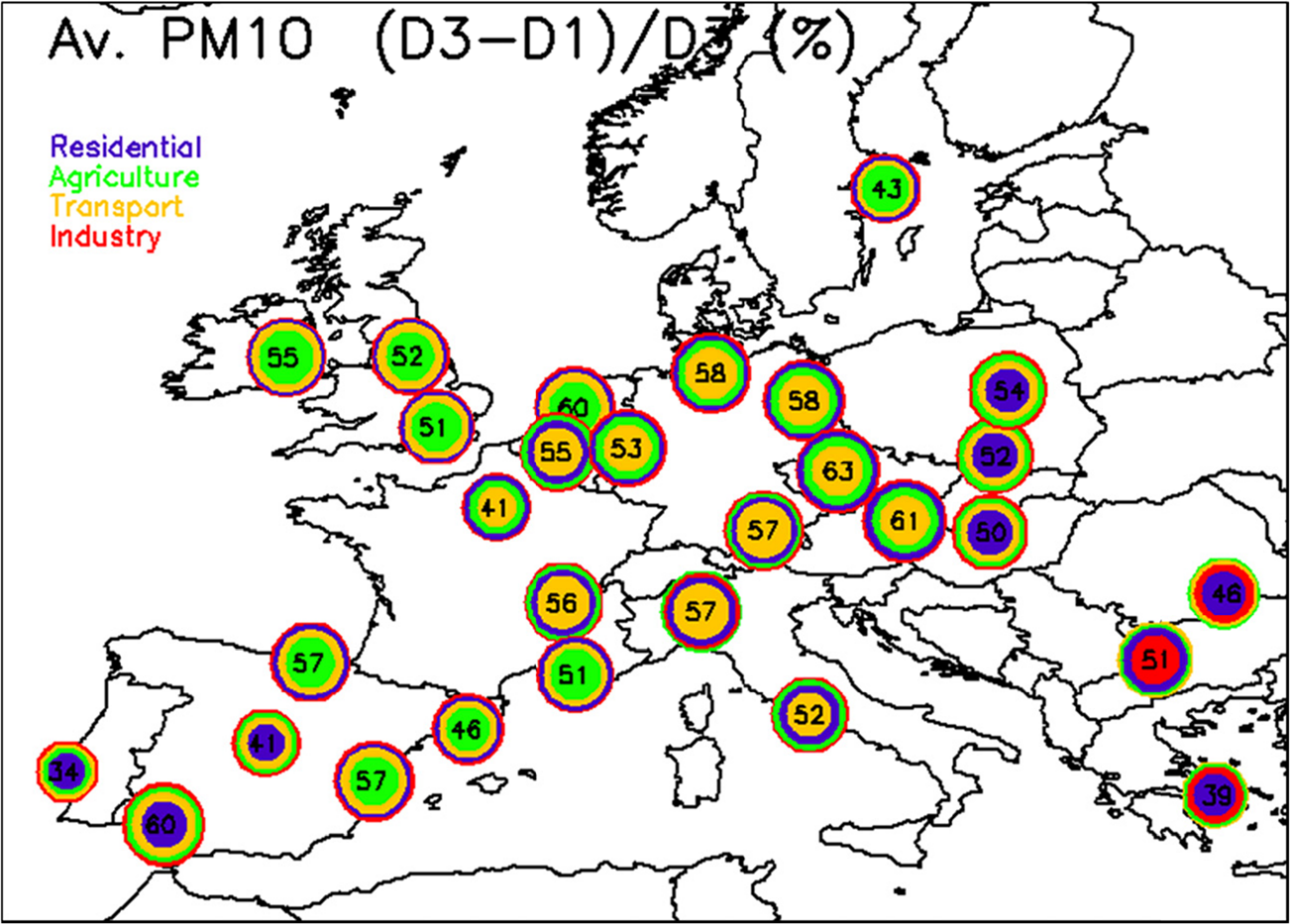
Same as Fig. 1 but shown for the relative change between *D*_AQP_ = 1 and *D*_AQP_ = 3 (in percentage). As an example, the Paris overall relative potential is 43 and 73 %, at *D*_AQP_ = 1 and *D*_AQP_ = 3, respectively. The relative change between *D*_AQP_ = 1 and *D*_AQP_ = 3 (expressed in terms of *D*_AQP_ = 3) is then 41 % as shown in this figure. The relative contribution of the four sectors to these 41 % is represented by the *colored circles* from center outward

The impact of emission reductions applied over a longer time period, 72 h (i.e., *D*_AQP_ = 3) in our case, will be seen over larger geographical areas with possible interactions between plans adopted in different cities. This city trans-boundary impact is reflected in Eq. 6 (term *B*) in which the background anthropogenic concentration accounts for the reduced levels resulting from neighboring cities air quality plans, whereas terms *A*, *C*, and *D* remain similar for 24 and 72 h. Figure 3 shows the relative potentials obtained for PM_10_ for *D*_AQP_ = 3 (after 72-h emission reductions) while Fig. 4 shows the relative potential increase between *D*_AQP_ = 3 and *D*_AQP_ = 1.

As expected, all cities see an increase in terms of relative potentials between 24 and 72-h emission abatement action plans. The color code provides information on the key activity sectors contributing to this efficiency increase (from more to less important outwards). In most cities, the magnitude of this increase is significant, pointing out to the added value of anticipating the set up of measures and of designing concerted action plans (in between cities). In some areas, however, an increase is seen between *D*_AQP_ = 3 and *D*_AQP_ = 1 but the overall magnitude of the potential remains low, probably pointing to missing anthropogenic emissions in our potential calculation (e.g., shipping for coastal cities), or to an important contribution of natural sources.

It is interesting to analyze these efficiency increases in terms of geographic locations. Traffic keeps being the key contributor in central Europe (from Benelux to Italy, including Germany), while agriculture remains the major contributor in the Northern part of Europe (UK, NL, and Stockholm) and in some parts of Spain, residential heating remains the key contributor in the Eastern countries and in the Iberian Peninsula. Industry, which was a minor contributor for short-term action plans, sees its importance growing in many cities due to the larger time allowed to diffuse emissions from stack height to surface and transport them towards the city centers to impact concentration. This is especially the case in the south-eastern cities like Sofia, Bucarest, or, to a lesser extent, Athens. Therefore, not only should action plans be concerted among cities but they also should target different sectors, depending on geographical specificities.

**Fig. 5:**
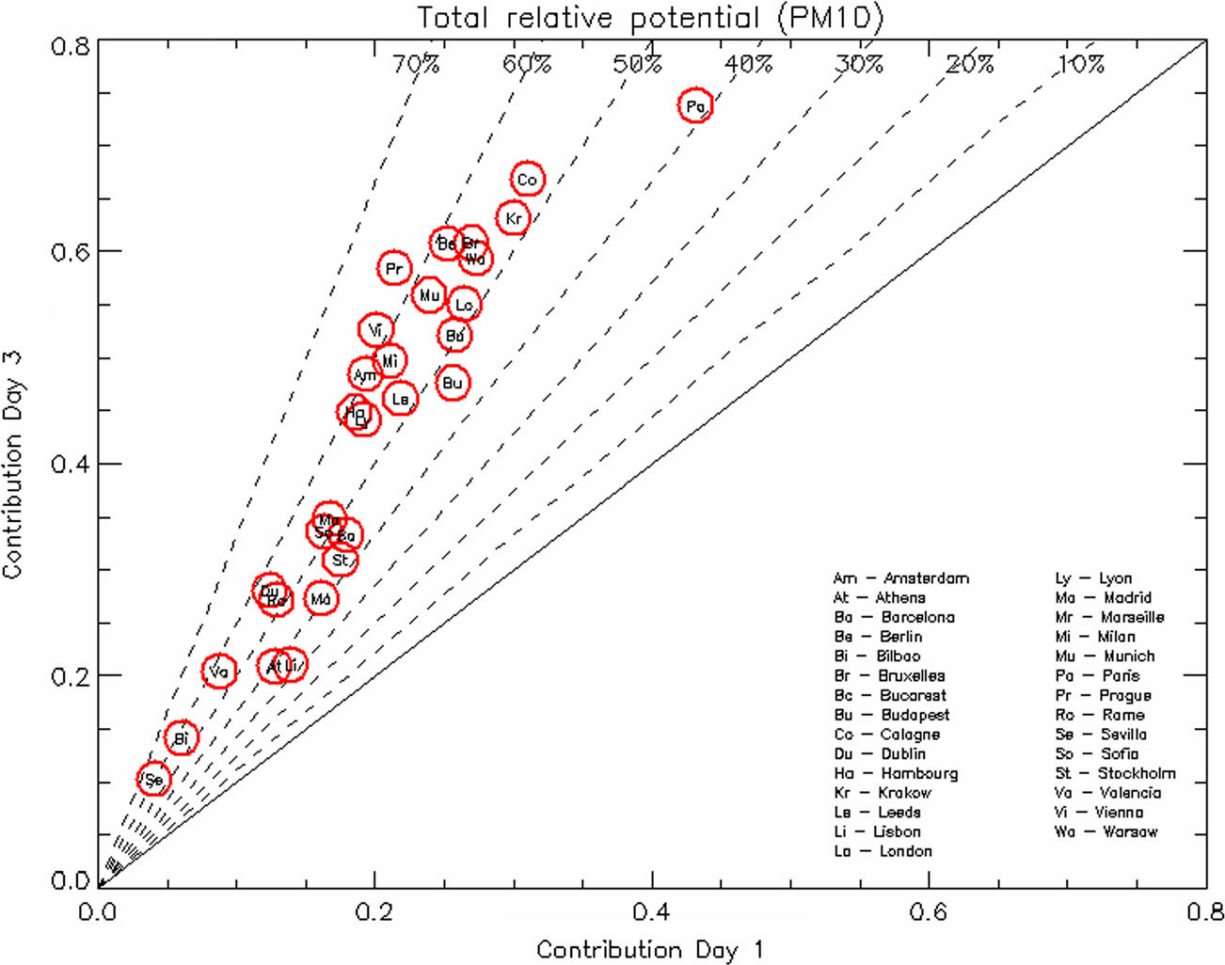
Relative potentials at *D*_AQP_ = 3 as a function of relative potentials at *D*_AQP_ = 1 for the series of cities analyzed. The *dashed lines* indicate the relative gain (with respect to *D*_AQP_ = 3) of maintaining the emission reductions (of all four sectors) over a duration of 3 days

A different view of these results is proposed in Fig. 5 where the relative potential reached for *D*_AQP_ = 3 is presented in terms of its value for *D*_AQP_ = 1. Dashed lines provide information on the percentage gain in efficiency when moving from a 24 to a 72-h time action plan. As mentioned earlier, the gain is significant for all cities and indicates the added value of multi-city concerted action plans (or regional and/or national action plans). We also note that the size (or importance) of a city is not correlated with the importance of the city trans-boundary impacts. Paris shows the highest potential but a moderate increase in efficiency whereas medium size cities like Prague or Vienne show moderate potentials but high increase in efficiency. Cities with low values include the coastal cities (Bilbao, Valencia, Athens, etc.) where other emission sources like shipping, dust and sea salt are not controlled in our analysis.

The case of Milan is interesting. This city exhibits a low potential value (21 %) for *D*_AQP_ = 1 but a large gain in efficiency for *D*_AQP_ = 3 (from 21 to 49 %, i.e., a percentage gain of 57 %) indicating the limited efficiency of plans constrained to Milan itself (or to its close neighborhood), and the need of extending these plans to the entire Po valley.

**Fig. 6:**
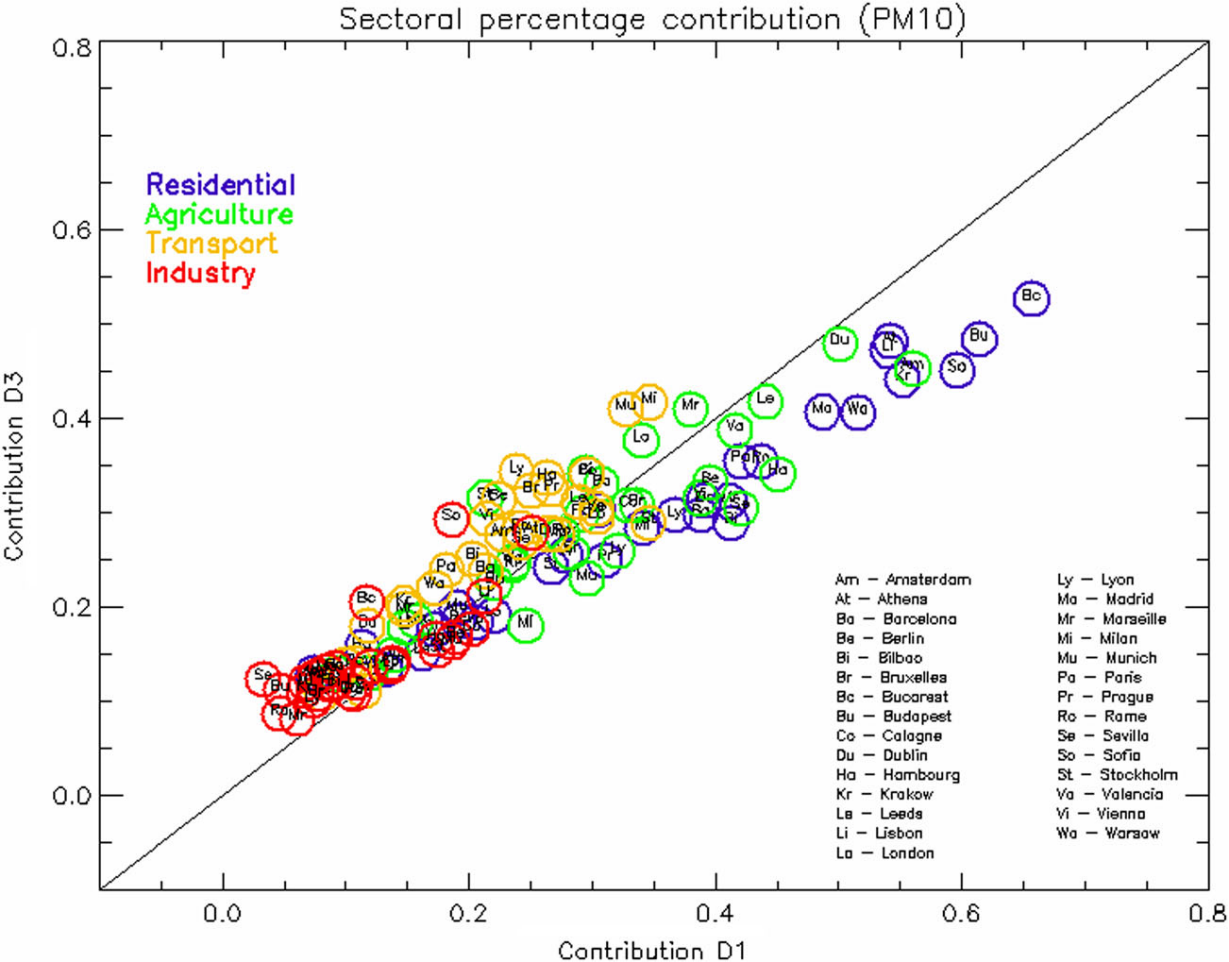
Same as Fig. 5 but with results detailed by sectors

In Fig. 6, the proportional contribution of each of the four activity sectors (TRA, RES, IND, and AGR) is shown for *D*_AQP_ = 1 and *D*_*AQP*_ = 3. Industry is the lowest contributor for all cities (with some exception like Sofia) but sees its importance growing when action plans are extended in time. As mentioned earlier, this is most probably due to the increased time allocated to stack releases to mix downwards and impact surface concentrations. Residential heating is by far the most important contributor in eastern countries but sees its relative importance decreasing with time. This is related to the increase of the transport contribution. Primary particulate matter arising from heating (in particular wood burning) have a more direct impact on concentration in a first stage, while the abundance of emissions from traffic reinforces this sector importance in a second stage.

**Fig. 7:**
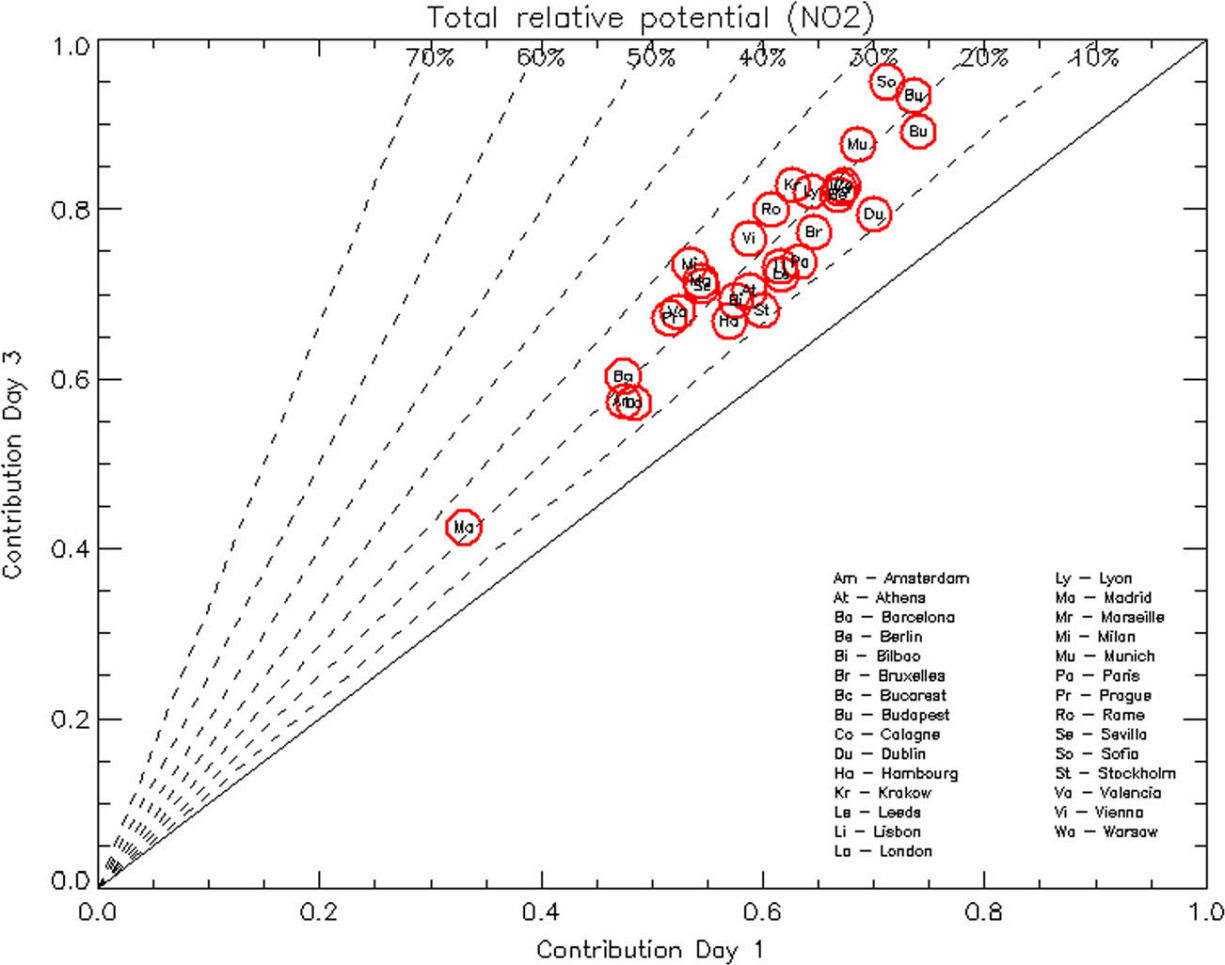
Same as Fig. 5 but for NO_2_

For NO_2_, the situation is quite different and the gain in efficiency between *D*_AQP_ = 1 and *D*_AQP_ = 3 is relatively limited (Fig. 7). Maximum impacts are of the order of 30 % for Milan with most of them lying around 20 %. The large potentials reached for *D*_AQP_ = 1 as well as the limited gain from *D*_AQP_ = 1 to *D*_AQP_ = 3 confirm the local character of the NO_2_ pollution and the little added value of city (or higher level) concerted actions to reduce the average concentrations for this pollutant. For most of the cities, the calculated potentials are high, showing that transport is by far the dominating sector, with the exception of Marseille where shipping and other sources most probably explain the very low value observed (around 35 %).

**Fig. 8:**
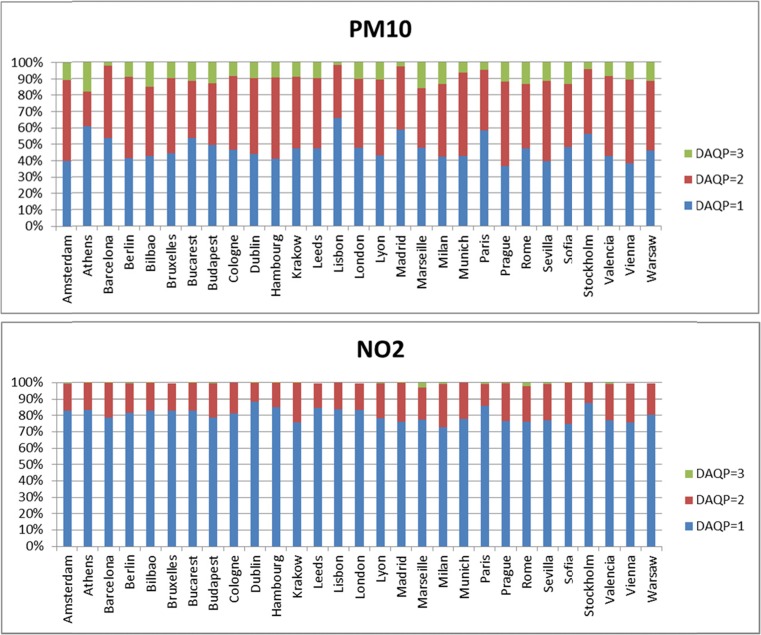
relative potential apportionment between *D*_AQP_ = 1 (*blue*), *D*_AQP_ = 2 (*red*), and *D*_AQP_ = 3 (*green*), for PM_10_ (*top*) and NO_2_ (*bottom*)

As a further analysis, the repartition of the efficiency gains between *D*_AQP_ = 1, *D*_AQP_ = 2, and *D*_AQP_ = 3 is illustrated in Fig. 8. For PM_10_, most of the gain is already obtained after 48 h; while for NO_2_, most of it is obtained after 24 h. But differences among cities obviously remain. We should also remember that the set of days is not continuous in time. The conclusions might change if a full-length long-lasting episode (i.e., lasting several days) is considered, with across scales impacts probably lasting more days.

## Implementation of short-term action plans

The scenarios designed in this analysis are all based on fixed emission reductions of 30 %, without suggesting how these emission reduction measures could be implemented in reality. As mentioned earlier, all results are assumed to be linear (between emission reductions and impacts) and a re-scaling of the results to the actual emission reduction level used in practice is straightforward. We provide in this section a few references to short-term air quality plans to put in perspective our results with a particular look at the intensity of the emission reductions and to their associated impacts.

Examples of STAPs mostly relate to applications in China to regulate air quality during specific international events. For example, Schleicher et al. (2012) discuss the evolution of atmospheric particles before, during, and after the period of the Olympic Summer Games in Beijing, China, in August 2008. This is done in order to investigate the efficiency of the mitigation measures implemented by the Chinese Government (Cermak and Knutti, 2009). The results showed that the applied aerosol source control measures (such as shutting down industries, reducing traffic, and increasing public transportation in a radius of about 150 km around Beijing) had a huge impact on the aerosol pollution in the city. However, the meteorological conditions, especially rainfall, certainly also contributed to the successful reduction of particulate air pollution. During the Olympic Games, the daily emissions of SO_2_, NO_x_, PM_10_, and NMVOC were 41, 47, 55, and 57 % lower than June 2008 emission levels (Wang et al., 2011). As a result, PM_2.5_ concentrations were reduced by roughly 47 % when compared to average concentrations of 12-h samples before the Olympic Games (Schleicher et al. 2012).

Liu et al. (2013) presented a similar work, but related to the Asian Games (12–27 November 2010) to analyze the relationship between emissions and concentrations of pollutants in Guangzhou. In addition to structural measures, during this period, additional measures were applied on (i) construction activities, which were placed on hold; (ii) odd/even restrictions for personal vehicle; and (iii) strict supervision of point sources to reduce emissions. The Asian Games abatement strategy totally reduced emissions of 41.1 % SO_2_, 41.9 % NO_x_, 26.5 % PM_10_, 25.8 % PM_2.5_, and 39.7 % VOC.

During the 2010 Expo in Shanghai, air quality improvement (Huang et al. 2013) was attributed to the municipal government, which implemented a series of control strategies, including long-term, region-wide, and short-term measures. Examples of short-term measures were related to power plants (phase out, adjust or relocation of heavily polluting industries and power plants; installation of flue gas desulphurization devices, etc.); transportation (promoting clean energy busses and taxis, access restriction of vehicles not compliant with emission standards, etc.); and construction work (establishment of smoke and dust control zone from downtown Shanghai to suburban areas; dust-prevention measures at construction sites, etc.).

In France, to reduce the impact of the severe PM episodes which occurred in March 2014 and 2015, a gradual set-up of measure was designed, from free public transportations, free car park to restricted domestic car traffic (car with odd or even number plate only authorized to circulate). More generally, an ensemble of measures has been designed targeting all activity sectors and according to the characteristics of the pollution episode, the expected most efficient measure(s) can be selected and set-up by local authorities to reduce the pollutant concentrations.

In a recent study, Janssen et al. (2010) assessed the impact of different abatement measures in Antwerp (Belgium): (a) implementation of a low emission zone in the city center, (b) closure of the ring road, and (c) the imposition of a speed limit reduction from 120 to 90 km/h on several major highways during particulate matter smog episodes. Based on quantitative model assessment, the authors conclude that the impact of traffic related mitigation strategies on PM_10_ and PM_2.5_ levels remain limited and only reach a few percent, although emission reductions are significant (of the order of 30 %).

As a final example of measures that can be applied in short-term action plans, a review of short-term action plans in Europe was presented by AEA (2012). The main measures used in Europe for short-term plans are related to: speed limitation, diesel driving restrictions, free public transport, intensification of periodic cleaning of streets (especially during long rainless periods), or to actions to reduce domestic heating emissions. However, the impact of these different measures was not assessed.

## Uncertainties and limitations of the approach

In this section, we come back to some of the main assumptions and limitations of the approach.

One main limitation resides in the non-controlled anthropogenic emission sources (e.g., shipping). They are referred to as “non-controlled” as no specific scenario has been designed to quantify their impact on concentration levels. The impact of those non-controlled emission sources cannot be distinguished from the natural and anthropogenic background. This also implies that the potential of human interventions is bigger than shown through the computed sectoral potentials. This becomes a limitation especially in locations where these non-controlled sources are expected to be important, in harbor cities (shipping) or within the Iberian Peninsula where dust events are frequent. Although this does not invalidate the conclusions drawn in the previous sections, it must be kept in mind when interpreting the results.

By definition, the relative potentials for *D*_AQP_ = 1, *D*_AQP_ = 2, and *D*_AQP_ = 3 represents averages over the 5-month period considered in this study. Given the incomplete time coverage within the 5-month period, the average potentials will therefore be based on different sets of points for their calculation. We therefore do not completely fulfill the conditions (described in the Annex) to ensure that meteorological effects are fully smoothed out during our analyses. However, the available days (spanning from January to May) cover high and low concentration days within each datasets, and differences in terms of average concentration levels remain low (see base case concentrations in Tables 2 and 3). The largest deviations are seen in the Spanish cities (Barcelona (10 %), Valencia (18 %)) and in some Eastern capitals (Bucarest, Warsaw) where the difference is a little less than 10 %.

**Table 2:** Relative potentials for all four activity sectors for PM10. The first columns for *D*AQP = 1 and *D*AQP = 3 give the base case average concentrations (μg/m3). The symbol *π* stands for the relative potential (Δ*C*/α*C*)

City	*D*_AQP_ = 1					*D*_AQP_ = 3				
	CBC	πAGR (%)	πIND (%)	πRES (%)	πTRA (%)	CBC	πAGR (%)	πIND (%)	πRES (%)	πTRA (%)
Amsterdam	16.6	11	3	1	4	17.0	22	7	6	13
Athens	45.1	1	3	7	1	43.5	3	6	10	2
Barcelona	20.7	6	2	7	4	22.9	11	4	10	8
Berlin	14.9	10	5	5	6	15.7	20	10	11	19
Bilbao	15.5	2	1	2	1	16.8	5	2	4	4
Bruxelles	21.6	8	2	11	7	21.9	16	7	19	20
Bucarest	22.9	3	3	17	3	25.0	6	10	25	7
Budapest	22.7	6	1	16	3	24.0	12	6	25	9
Cologne	18.4	10	6	5	9	18.5	21	12	12	23
Dublin	9.5	6	1	2	3	9.8	13	3	4	8
Hambourg	16.7	8	3	2	5	17.7	15	7	7	15
Krakow	20.3	7	2	17	4	20.7	15	7	28	13
Leeds	11.1	10	2	4	6	1.9	19	6	7	14
Lisbon	18.8	2	3	7	1	20.3	4	4	10	3
London	18.1	9	4	6	8	17.5	21	8	11	16
Lyon	18.0	6	1	7	5	18.8	11	4	13	15
Madrid	19.0	3	1	8	4	19.9	5	4	11	8
Marseille	19.7	6	1	7	2	18.6	14	3	11	7
Milan	31.1	5	1	7	7	31.5	9	6	14	21
Munich	13.8	7	4	5	8	14.5	13	9	11	23
Paris	24.1	13	5	18	8	25.3	22	8	26	18
Prague	12.3	7	2	7	6	12.3	18	7	15	19
Rome	34.2	4	1	6	3	31.9	8	2	10	8
Sevilla	24.3	2	0	1	1	26.2	3	1	3	3
Sofia	23.3	2	3	10	1	24.3	5	10	15	4
Stockholm	8.6	4	3	5	6	8.9	10	5	7	9
Valencia	14.7	4	1	2	3	17.2	8	3	4	6
Vienna	14.5	8	2	6	4	14.9	17	7	13	15
Warsaw	16.8	6	2	14	5	18.4	15	8	24	13

**Table 3:** Same as Table 1 but for NO_2_

City	*D*_AQP_ = 1					*D*_AQP_ = 3				
	CBC	πAGR (%)	πIND (%)	πRES (%)	πTRA (%)	CBC	πAGR (%)	πIND (%)	πRES (%)	πTRA (%)
Amsterdam	13.2	1	7	12	28	14.1	0	9	15	34
Athens	13.8	0	5	19	34	14.6	−1	6	24	41
Barcelona	24.0	0	10	6	32	24.0	−1	13	8	40
Berlin	10.2	0	6	18	43	10.5	−1	8	22	53
Bilbao	4.2	0	12	7	39	4.2	−1	15	9	46
Bruxelles	19.9	0	11	12	42	20.8	−1	13	14	50
Bucarest	9.6	0	9	13	52	10.0	0	12	16	62
Budapest	9.3	0	7	23	44	9.3	−1	10	29	56
Cologne	19.3	0	5	12	50	18.7	−1	7	16	61
Dublin	3.2	0	11	13	46	3.1	−1	12	15	53
Hambourg	11.0	0	4	11	41	12.2	0	5	14	48
Krakow	10.9	0	7	18	38	10.7	−1	10	24	50
Leeds	9.8	0	10	11	42	9.9	−1	11	12	49
Lisbon	6.0	0	14	6	41	6.1	0	17	7	50
London	25.8	0	8	10	31	26.7	−1	10	11	37
Lyon	8.5	0	6	14	45	8.4	−1	7	18	57
Madrid	22.6	0	10	7	38	22.8	−1	13	9	50
Marseille	16.0	−1	3	8	22	14.6	−1	4	10	29
Milan	29.5	0	5	9	40	28.2	−1	7	13	54
Munich	10.8	0	5	13	51	11.1	0	6	17	64
Paris	24.5	0	8	16	39	25.0	−1	10	19	46
Prague	7.6	0	9	9	34	7.5	−1	12	12	44
Rome	18.6	0	6	15	40	17.2	−1	8	21	52
Sevilla	8.3	0	10	9	36	8.3	0	13	12	46
Sofia	4.3	0	22	7	42	4.3	0	32	10	54
Stockholm	3.8	0	18	7	35	4.0	0	21	8	39
Valencia	11.6	0	9	5	38	11.9	−1	12	7	50
Vienna	8.7	0	8	10	40	8.4	0	11	13	52
Warsaw	9.5	0	8	19	40	9.9	−1	11	24	49

One of the reasons brought forward to explain the regional differences in terms of relative potential (i.e., different magnitude, different key activity sector) is the underlying emission inventory. By definition, the potential indicator value is determined by the efficiency of the process (change in concentration resulting from a unit emission change) but also by the available amount of emissions. The conclusions reached here therefore strongly depend on the quality of the underlying emission inventory. The issues raised in a previous work (EC4MACS, Terrenoire et al. 2015) regarding the distribution of wood burning emissions in French urban areas is a good example. Overestimated wood burning emissions in city centers will directly lead to overestimated potentials. In this context, the methodology presented in this work could also be useful to detect possible inconsistencies and support the improvement of the underlying emission inventories. Indeed differences in relative potentials between simulations performed with different emission inventories allow identifying the main inconsistencies (which sectors, which areas, etc.) that need to be solved in priority.

The spatial resolution used for these simulations is coarse (~50 km) and is insufficient to capture correctly urban effects (Schaap et al. 2015), but it can be used to assess background trends. To improve the accuracy of the concentration levels, specific parameterizations have been developed. In this work, we rather focus on potentials which by construction (∆*C*/*αC* ) are less sensitive to urban increments (i.e., *delta* concentration correlate with concentration levels). In their sensitivity analysis, (Lauwaet et al. 2013) showed that the average potentials obtained with different spatial resolutions were comparable.

Our analysis is based on a linear assumption. Firstly, we assumed that each single precursor reduction would be linear (i.e., ∆^100%^ ≈ ∆^*α*%^/*α*); and secondly, interactions between precursors are assumed to be negligible (*f*_NL_ = 0). While these non-linear terms cannot be neglected on a daily basis, potentials calculated for longer term averages (e.g., seasonal) have been shown by Thunis et al. (2015) to behave mostly linearly. Given the incomplete time coverage and the final 2 months averages performed in this study, non-linearities might need to better be accounted for.

## Conclusions

Countries and regions have the legal obligation to design air quality plans and assess their impacts when concentration levels exceed the limit values. Because these limit values cover both short- (day) and long-term (month/year) effects, air quality plans also follow these two formats. While many integrated assessment tools focus on the long-term impacts, fewer have devoted their attention to assess the impact of short-term measures.

In this work the methodologies developed in the frame of the FAIRMODE network, in particular those designed to address the evaluation of responses of models to emission reduction scenarios, are used to further analyze MACC-III project simulations. The used scenarios, which specifically look at the residential, transport, agricultural, and industrial sectors, are produced on a daily basis with the aim of supporting policy makers in assessing the impact of short-term action plans.

Regarding PM_10_, the short-term results highlighted the diversity of responses across European cities, not only in terms of magnitude but also in terms of type (key sector contributing). This raises the necessity of designing plans which address the specificity of the area. Action plans extended to 3 days highlight the added value that trans-city (or regional and/or national) coordinated actions would have because of impacts of one city’s action plan on others. The largest benefits were seen to occur in central Europe (Vienna, Prague) while major cities (e.g., Paris) already solve a large part of the problem on their own. It is worth noting that coordination of action plans should account for the local/regional specificities. Eastern Europe would particularly benefit from plans based on emission reduction in the residential sectors while in northern cities, agriculture seems to be the key sector on which to focus attention. Transport is playing a key role in most cities whereas the impact of industry is limited to a few cities in south-eastern Europe.

For NO_2_, the situation is different. 24 h action plans focusing on traffic emission reductions are efficient in all cities. This is due to the more local character of this type of pollution as well as to the fact that the chemical transformations at the basis of the NO_2_ formation are rapid and therefore less dependent of transport. In a future study, the impact of the assumptions taken in this study (anthropogenic emission reductions not considered, temporal/spatial resolution of the model, data availability and linearity) will be further analyzed.

## Annex

This annex shows how the impact of varying meteorological conditions on the concentration change (when comparing *D*_AQP_ = 1 with *D*_AQP_ = 2 or *D*_AQP_ = 3 forecasts obtained on different days) is shown to be smoothed out when average values (based on a sufficient long time series) are considered. We will show it here for *D*_AQP_ = 2 but the same approach can be used to generalize this to *D*_AQP_ = 3. With slightly simplified notations, Eq. (1) can be re-written as follows for *D*_AQP_ = 2:

7 $$ {C}^i={C}_{bg}^i+{\displaystyle \sum_{d=-\infty}^{i-2}\varDelta {C}^d}+\varDelta {C}^{i-1}+\varDelta {C}^i $$

**Fig. 9:**
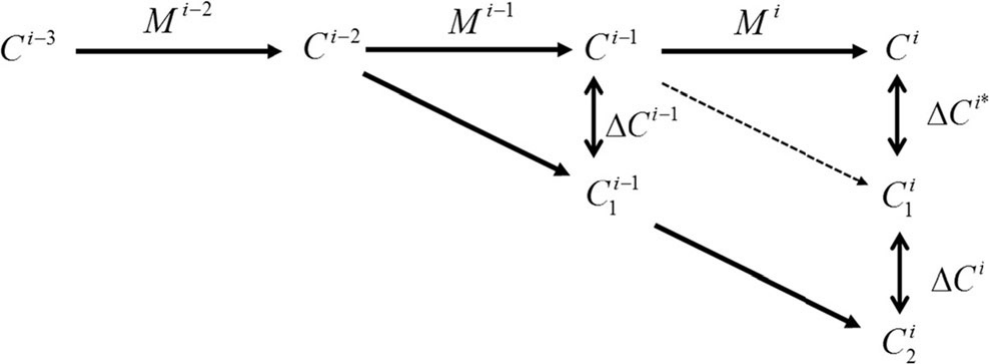
Schematic representations of the concentration forecast (*C*) and underlying meteorology (*M*) over a 3-day period. The superscript is indicative of the forecasted day whereas the subscript indicates the duration of the emission reduction. This diagram supports the analysis made in the Annex. The *dashed line* indicates a pathway not covered by a model scenario

Figure 9 represents graphically some of the terms of this equation. We see that we need to assume:

8 $$ \varDelta {C}^{i-1}=\varDelta {C}^{i*} $$

to write Eq. (7). This is obviously not the case for a single day because the meteorology could vary and impact the resulting concentration change obtained by reducing emissions by the same amount on two different days. Let us now consider averaging these terms over the number of available days (55 in our case). From the available model scenarios (base case, 1- and 2-day-long emission reductions) only *ΔC* ^*i* − 1^ and the sum *ΔC* ^*i*^ + *ΔC* ^i*^can be estimated directly (Fig. 9). Averaged, we can rewrite the two terms in Eq. (8) as:

9 $$ \overline{\varDelta {C}^{i-1}}=\overline{C^{i-1}}-\overline{C_1^{i-1}}\kern0.5em \mathrm{and}\kern0.5em \overline{\varDelta {C}^{i*}}=\overline{C^i}-\overline{C_1^i} $$

The two terms $\overline{\varDelta {C}^{i-1}}$ and $\overline{\varDelta {C}^{i*}}$ are equal if:

$$ \overline{C^{i-1}}=\overline{C^i}\kern0.5em \mathrm{and}\ \overline{C_1^i}=\overline{C_1^{i-1}} $$

or, in other words, if the averaged concentration for the base case or for the 1-day-long emission reductions are similar. The validity of this assumption can be assessed by consulting Table 1 where the average base case concentrations used at *D*_AQP_ = 1 and *D*_AQP_ = 3 are compared. As can be seen from this table, the differences remain in general low, with the exception of a couple of cities, and therefore validate the approach. This is why all equations in this work refer to average concentrations.
